# Development of a POC Test for TB Based on Multiple Immunodominant Epitopes of *M. tuberculosis* Specific Cell-Wall Proteins

**DOI:** 10.1371/journal.pone.0106279

**Published:** 2014-09-23

**Authors:** Jesus M. Gonzalez, Bryan Francis, Sherri Burda, Kaitlyn Hess, Digamber Behera, Dheeraj Gupta, Ashutosh Nath Agarwal, Indu Verma, Ajoy Verma, Vithal Prasad Myneedu, Sam Niedbala, Suman Laal

**Affiliations:** 1 Department of Chemistry, Lehigh University, Bethlehem, Pennsylvania, United States of America; 2 TB Biosciences, Bethlehem, Pennsylvania, United States of America; 3 Department of Pathology, New York University Langone Medical Center, New York, NewYork, United States of America; 4 Postgraduate Institute of Medical Education and Research, Chandigarh, India; 5 National Institute of Tuberculosis and Respiratory Diseases, New Delhi, India; 6 Veterans Affairs New York Harbor Healthcare System, New York, New York, United States of America; Université de Montpellier 2, France

## Abstract

The need for an accurate, rapid, simple and affordable point-of-care (POC) test for Tuberculosis (TB) that can be implemented in microscopy centers and other peripheral health-care settings in the TB-endemic countries remains unmet. This manuscript describes preliminary results of a new prototype rapid lateral flow TB test based on detection of antibodies to immunodominant epitopes (peptides) derived from carefully selected, highly immunogenic M. tuberculosis cell-wall proteins. Peptide selection was initially based on recognition by antibodies in sera from TB patients but not in PPD-/PPD+/BCG-vaccinated individuals from TB-endemic settings. The peptides were conjugated to BSA; the purified peptide-BSA conjugates striped onto nitrocellulose membrane and adsorbed onto colloidal gold particles to devise the prototype test, and evaluated for reactivity with sera from 3 PPD-, 29 PPD+, 15 PPD-unknown healthy subjects, 10 patients with non-TB lung disease and 124 smear-positive TB patients. The assay parameters were adjusted to determine positive/negative status within 15 minutes via visual or instrumented assessment. There was minimal or no reactivity of sera from non-TB subjects with the striped BSA-peptides demonstrating the lack of anti-peptide antibodies in subjects with latent TB and/or BCG vaccination. Sera from most TB patients demonstrated reactivity with one or more peptides. The sensitivity of antibody detection ranged from 28–85% with the 9 BSA-peptides. Three peptides were further evaluated with sera from 400 subjects, including additional PPD-/PPD+/PPD-unknown healthy contacts, close hospital contacts and household contacts of untreated TB patients, patients with non-TB lung disease, and HIV+TB- patients. Combination of the 3 peptides provided sensitivity and specificity>90%. While the final fully optimized lateral flow POC test for TB is under development, these preliminary results demonstrate that an antibody-detection based rapid POC lateral flow test based on select combinations of immunodominant M. tb-specific epitopes may potentially replace microscopy for TB diagnosis in TB-endemic settings.

## Introduction

Over 90% of the estimated>9 ×10^6^ new cases of TB occur in developing countries where clinical suspicion, microscopic examination of smears made directly from the sputum samples for acid fast bacilli (AFB), and occasionally chest X-rays remain the methods of choice for TB diagnosis. Microscopy is tedious, time-consuming, requires examination of multiple specimens and fails to identify paucibacillary patients (sputum smear-negative, extrapulmonary TB (EPTB) patients). However, the high patient burden and limited resources allow the TB control programs in the endemic countries to focus only on detection and treatment of highly infectious TB cases [1]. In contrast, in settings with ample resources and low patient burdens, TB diagnosis is based on smears made from decontaminated and concentrated specimens, nucleic acid-amplification tests (NAAT) and culture of bacteria from patient specimens. While these technologies are more sensitive than the direct sputum smear, the required lab infrastructure, trained personnel and high patient-burden makes their implementation in TB-endemic settings impractical. A new automated NAAT test, the gene-Xpert (GXP) which is highly sensitive and specific, and requires minimal training, has been endorsed by the WHO as a diagnostic tool [2]. However, the cost of the instrument, need for regular maintenance and calibration, limited throughput, the requirement for ambient temperatures (15–30°C) which needs air-conditioning, and the expensive cartridges make it difficult to implement the GXP as a POC test in most TB-endemic settings [3], [4]. The global need for a rapid, robust, inexpensive point-of-care (POC) TB test that can be implemented in the microscopy centers of the TB control programs and in other peripheral health care settings remains unmet [5].

## Materials and Methods

### Study populations

Data reported in this manuscript are based on banked serum specimens, a vast majority of which were obtained over several years from subjects.

#### TB Patients

Sera were obtained from 104 AFB smear positive TB patients recruited at the National Institute of Tuberculosis and Respiratory Diseases (NITRD; formerly the Lala Ram Sarup Institute of Tuberculosis and Respiratory diseases), New Delhi, India and the Post Graduate Institute for Medical Education and Research (PGIMER), Chandigarh, India. Subjects were recruited after obtaining approvals from the NITRD Ethics Committee and the PGIMER Ethics Committee. Hard copies of the informed consent forms were either signed by, or the thumb impression obtained from each individual recruited. Fourteen of the 104 smear positive TB patients were co-infected with HIV, (CD4+ T-cell range 161–763 cells/mm^3^, 2 unknown), the viral loads were not known. Sera from 10 HIV- smear-positive TB patients from South Africa were kindly provided by Dr. William Rom, Bellevue Hospital, NY, NY; these specimens were collected after necessary approvals from the New York University Langone Medical Center Institutional Review Board and informed, written consents were collected from each individual.

#### Patients with NTBLD

Sera from 26 NTBLD patients were obtained from PGIMER. These included 16 patients with sarcoidosis diagnosed on the basis of presence of clinical features of pulmonary involvement and consistent radiological involvement, presence of compact non-caseating granulomas and absence of Acid fast Bacilli in transbronchial lung biopsy, and good clinical response to steroids without ATT. Five patients with lung cancer (two of whom had malignant cells in their pleural effusion), 1 renal failure patient with pleural effusion, 1 patient with allergic bronchial aspergillosis and 2 SLE patients with pulmonary involvement, and 1 patient with pemphigous pneumonia. The diagnosis of the 10 non-sarcoid NTBLD patients was based on clinical/radiological/cytological/histopathology proven criteria for the respective conditions; no specific efforts to rule out TB were made for these patients.

#### PPD+, PPD-, PPD-unknown, HC and HHC, and HIV+TB- subjects

As shown in Table 1, Sera from 50 hospital employees with routine close contact with TB patients (healthy contacts), 27 PPD-, 22 PPD+ and 15 PPD-unknown healthy subjects were also obtained from PGIMER and NITRD. No additional investigations for TB were done in these subjects. Sera from 30 household contacts of untreated AFB smear positive TB was obtained from NITRD. TB was ruled out in these contacts on the basis of clinical examination and chest X-rays. In addition, sera from 72 HIV+TB- subjects on ART were kindly provided by Dr. Susan Zolla-Pazner, New York University Langone Medical Center (NYULMC), NY, NY; these specimens were collected after necessary approvals from the Veterans Affairs New York Harbor Healthcare System Institutional Review Board and informed, written consents were collected from each individual. These individuals from the US are at a very low risk for TB and no investigations for TB were done. The remaining PPD+ or PPD- healthy subjects whose sera were included in these studies were recent immigrants from TB-endemic countries working at NYULMC and were bled after obtaining written informed consent.

**Table 1:** Clinical characteristics of study subjects.

TB status	HIV Status	Other	Subjects, no.	Geographic Region	Sputum Smear	Treatment†
Positive	Negative	-	100	India	Positive	Untreated
		-	10	South Africa	Positive	Untreated
Positive	Positive	-	14	India	Positive	Untreated
Negative	Positive	-	72	United States	ND	ART
Negative	Negative	Lung Disease	26	India	ND	NA
Negative	Negative	Household Contact	30	India	ND	NA
Negative	Negative	Healthy Contact	50	India	ND	NA
Negative	Negative	PPD Negative	5	United States	ND	NA
			27	India	ND	NA
			4	China	ND	NA
			1	Germany	ND	NA
			1	Indonesia	ND	NA
			1	Jamaica	ND	NA
			1	Romania	ND	NA
			1	Vietnam	ND	NA
Negative	Negative	PPD Positive	5	United States	ND	NA
			22	India	ND	NA
			8	China	ND	NA
			3	Cameroon	ND	NA
			2	Poland	ND	NA
			1	Columbia	ND	NA
			1	Spain	ND	NA
Unknown	Negative	PPD Unknown	15	India	ND	NA

† Treatment status at the time of serum collection; ND: Not done; ART: Anti-retroviral therapy; NA: Not applicable.

Each serum specimen was aliquoted and stored at −80°C. For use in experiments, 20 µls of each serum specimen was combined with 2 µl of a 10% Triton X-100/1× phosphate buffered saline (PBS) solution and incubated for 30 min at room temperature to inactivate any viruses. Subsequently the serum specimens were diluted 1∶10 with 1XPBS containing 2.5% bovine serum albumin, aliquoted and stored at −20°C.

### Development of Lateral Flow POC test for TB

The POC lateral flow test reported here is based on detection of serum antibodies directed against selected immunodominant epitopes of highly immunogenic cell-wall proteins of *M. tb* [6]–[8]. The antibodies are captured between BSA-peptide conjugates that are immobilized onto gold particles and the same peptide immobilized on the lateral flow strips. The intensity of the test line is directly proportional to the level of serum antibodies against the target peptide The following sections describe the construction of the components of the lateral flow test and their performance with banked sera from various types of TB patients and non-TB controls.

### Selection of Peptides

The identification of immunodominant epitopes of 2 cell-wall proteins of *M. tb*, PTRP (Rv0538) and LipC (Rv0220) by epitope mapping by ELISA was previously reported [7], [8]. Briefly, overlapping peptides, each with a biotin residue attached to the N-terminal, and covering the sequence of the two proteins were synthesized commercially. Individual peptides were captured in wells of streptavidin-coated plates (StreptaWell, Roche Diagnositcs, Germany) and tested for reactivity with sera from 36 PPD-/PPD+ healthy subjects and 60 smear-positive TB patients. Peptides that showed minimal or no reactivity with sera from PPD- and PPD+ subjects, and were individually recognized by 40% or more of the 60 TB patients (not included in the current studies) whose sera were used for epitope mapping were designated as immunodominant epitopes [7]–[9]. Similarly, epitope mapping using overlapping peptides representing another highly immunogenic cell-wall protein identified earlier, PE-PGRS51 (Rv3367) was conducted (Unpublished). A total of 33 immunodominant peptides were identified, 9 of which designated P1-P9 are included in the current report.

### Preparation of peptide-BSA conjugates

An EDC/NHS two-step protocol was used to conjugate each of the 9 peptides blocked at the N-terminus to BSA (Sigma-Aldrich). Each peptide was dissolved in 50 µL of DMSO (Fisher Scientific) and mixed with a 4× molar ratio of NHS (Sigma), EDC (Pierce). The activated peptide ester solution was added to a 40 mg/mL BSA solution on ice in 50 mM HEPES pH 7.4. After the coupling reaction was allowed to incubate overnight, the solution was dialyzed against 50 mM HEPES pH 7.4 for 72 hours with seven changes of buffer. Post dialysis, the purified conjugates were analyzed by MALDI-TOF mass spectrometry (Bruker Daltonic LTD., ON, Canada) to ensure modification [10].

### Preparation of lateral flow strips

To immobilize each BSA-peptide conjugate on a test line, various concentrations of BSA-peptide conjugates were diluted in 100 mM Sodium Carbonate pH 9.65 (final concentration 0.1-1.0 mg/mL). The test line solution was applied to the nitrocellulose membrane (190 × 25 mm) at 2 µl/cm using a CAMAG Linomat 5 dispenser (CAMAG Scientific, Wilmington, NC). The prepared membranes were dried at 35°C in a Thermo-Scientific incubator (Fisher Scientific, Pittsburg, PA) for 48 hours, after which the membranes were removed from the incubator and stored at ambient temperature and 20% relative humidity in a Dry Keeper dessicator cabinet with a dial hygrometer (Bel Art Products, Pequannick, NJ). The dry nitrocellulose membranes, along with the absorbent and sample pads were assembled onto backing cards using a Kinematic Matrix 2210 universal card laminator (Kinematic Automation, Twain Harte, CA) and cut into 3.8 mm strips using Azco Sur Size automatic guillotine cutter model SS4 (AZCO, Elmwood Park, NJ) [11].

### Gold conjugate preparation

Various concentrations (10 ug/mL–100 ug/mL) of BSA-peptide conjugates were diluted in 50 mM HEPES pH 7.2 and mixed with sufficient 40 nM colloidal gold nanoparticles to attain an OD_540 nm_ 1.5 (Arista Biologicals, Allentown, PA). The solutions were incubated overnight at ambient temperature followed by centrifugation at 12000xg for 10 minutes. The gold particles were resuspended in the same buffer, centrifuged three times, and the supernatant discarded. After centrifugation the gold conjugates were stored in HEPES pH 7.2 [12].

### Optimization of reactivity of BSA-peptide conjugates with sera in Lateral Flow Format

To determine the optimal conditions that discriminate specimens from TB patients and non-TB controls, 50 µls of serum pools prepared with sera from 5 PPD+ healthy subjects and 10 TB patients were diluted between 1∶100 and 1∶1000 in 50 mM HEPES pH 7.2, and mixed with 50 µls of various dilutions of peptide-gold conjugates. All 100 µls of the serum/peptide-gold mixture was applied to the lateral flow strips corresponding to the appropriate BSA-peptide test line. While the goal is to devise a lateral flow test that can be interpreted visually, the prototype being developed was interpreted visually after 15 minutes and also interrogated using an Avago Technologies Reader (San Jose California).

All subsequent experiments were performed with the sample dilution and peptide-gold conjugate concentrations that resulted in the highest signal with the TB pool sample while maintaining the PPD pool line below visually detectable levels.

### Evaluation of reactivity of BSA-peptides with sera from TB patients and non-TB controls

Following the optimization of serum/gold concentrations for each peptide, reactivity of all 9 BSA-peptide conjugates was evaluated with sera from 47 non-TB, 10 NTBLD and 124 TB patients. Sera were diluted to predetermined appropriate dilutions (for each peptide) in running buffer (2% BSA, 0.5% Tryptone, 1% Tween-20), and mixed with sufficient volume of peptide-gold-conjugate (OD_540 nm_ 30.0) to obtain the optimal concentrations determined in titration experiments above. The mixture was immediately added into the sample port of the lateral flow device. Photographs were taken at regular intervals and the intensity of the test line was read after 15 minutes. The mean reflectance obtained with sera from the 47 PPD-/PPD+/PPD-unknown samples plus 2.5 standard deviations was used as cut-off to determine positive or negative reactivity in sera from the different cohorts of subjects being included for test development. Three of the 9 peptide-conjugates were further evaluated for reactivity with sera from an expanded cohort of 400 subjects.

### Statistical Analysis

Data were analyzed using Graphpad prism v5 (Graphpad Software, San Diego, CA, USA). Reflectance values for healthy controls were compared with those for smear-positive TB patients, as were data for patients with NTBLD. The two-tailed Mann-Whitney test (95% CI) was used for analysis of statistical significance (P Value). The receiver operating characteristics (ROC) plots for assessing the ability of individual peptides and peptide combinations to distinguish between Sputum smear-positive TB patients from PPD-/PPD+/PPD-unknown subjects, and the same TB patients and close/household contacts/patients with NTBLD/HIV+TB- patients were plotted using the statistical package SPSS (version 22; SPSS Inc, Chicago, IL, USA). Calculation of sensitivity and specificity (with 95% CI) was performed by using Stata 2012 statistical software (Stata Corporation, Release 12, College Station, TX, USA).

## Results

### Reactivity of peptide-BSA conjugates with sera on lateral flow

**Figure 1:**
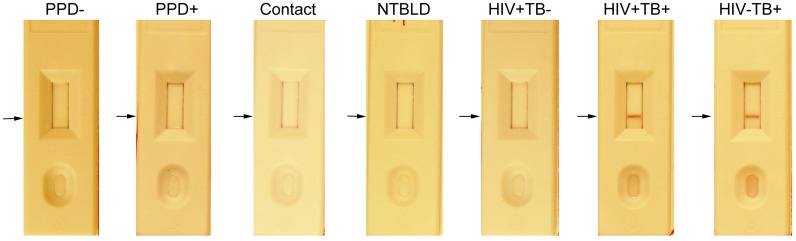
Visual example of reactivity of BSA-peptide P6 with sera on lateral flow. Reactivity with one serum specimen from each of the groups of non-TB controls and TB patients included in the study with BSA-peptide conjugate P6 is shown. In general the serum specimens from non-TB subjects exhibited no detectable reactivity with the striped BSA-peptide conjugate while sera from smear positive TB patients produced test lines easily distinguished by the eye.

**Figure 2:**
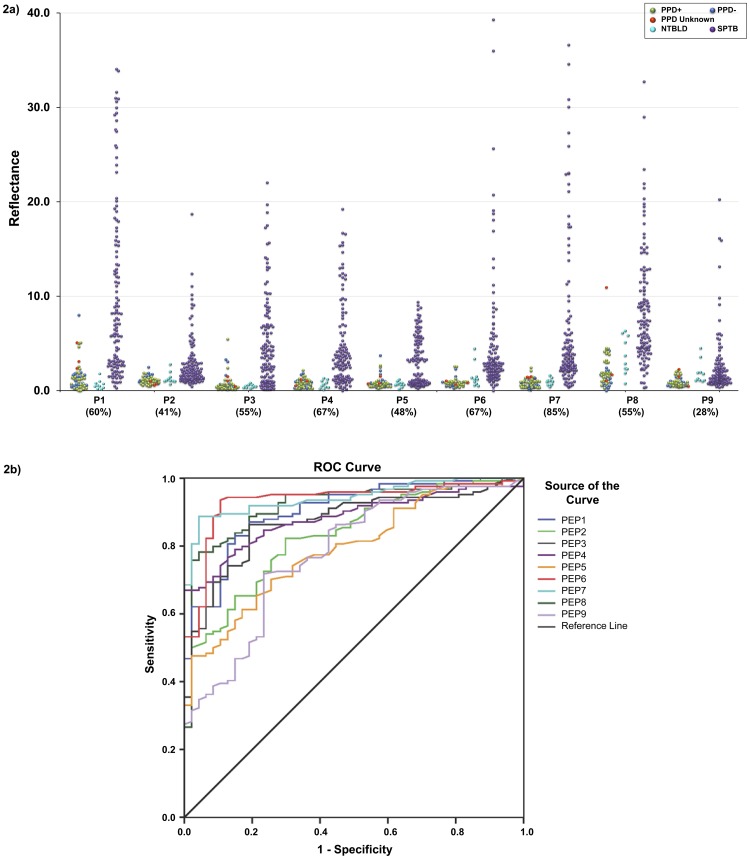
Reactivity of sera with individual BSA-peptide conjugates in lateral flow format. (A) Dot plot of reflectance values obtained with sera from healthy subjects [PPD- (blue), PPD+ (light green), PPD unknown (red); n = 47], non-TB lung disease patients [NTBLD (light blue); n = 10], and sputum smear-positive TB patients [SPTB (purple); n = 124, including 14 patients co-infected with HIV] tested for reactivity to BSA-peptide conjugates on lateral flow are plotted. Dashed lines indicate mean reflectance of the 47 healthy subjects plus 2.5 times the standard deviation which was used as cut-off to determine sensitivity in TB patients. Values in parenthesis under dot-plot for each BSA-peptide represent percent sensitivity of anti-peptide antibody detection obtained in TB patients with that peptide. The difference between healthy controls and TB patients was highly statistically significant (P<0.0001) for all 9 peptides. The P values for difference between the NTBLD and TB patients ranged from 0.0073−<0.0001 for P1-P8; for P9 the P value was 0.7221. (B) ROC curves representing the performance of the 9 BSA-peptide conjugates (P1–P9) on lateral flow format for reactivity with sera from healthy subjects and smear-positive TB patients.

As explained above, the reactivity of the different amounts of striped BSA-peptide conjugates on lateral flow format was tested with several dilutions of pooled sera from 5 PPD+ healthy subjects and 10 TB patients mixed with different dilutions of the peptide-gold conjugates, to determine the optimal conditions under which there was little to no background signal with PPD+ pool, and a strong signal was obtained with the TB serum pool (data not shown). A line which provides a reflectance value of 2 or less on the Avago Reader cannot be seen visually. Fig 1 depicts the reactivity of a BSA-peptide conjugate striped on nitrocellulose and probed with serum specimens from different classes of non-TB individuals and TB patients. The reactivity of sera from 47 non-TB healthy individuals (3 PPD-, 29 PPD+, 15 PPD-unknown), 10 patients with NTBLD and 124 sputum smear-positive TB patients with the 9 peptides on LF is depicted in Fig 2A. There was either no signal or a low signal obtained with a vast majority of sera from PPD-/PPD+/PPD-unknown healthy subjects for any of the peptides. In contrast, sera from TB patients demonstrated significantly stronger reactivity with the 9 BSA-peptide conjugates. The mean reflectance of sera from the 47 PPD-/PPD+/PPD-unknown subjects plus 2.5 SD obtained with any BSA-peptide conjugate was used as cut-off to determine sensitivity of antibody detection in the sera from the 124 TB patients with that BSA-peptide conjugate. The sensitivity of antibody detection ranged from 28% with P9, to 85% with P7 (Fig 2A). The difference between healthy controls and TB patients was highly statistically significant (P<0.0001) for all 9 peptides. ROC curves for the 9 peptides for TB patients with the control group as reference were plotted (Fig 2B). The AUC for the 9 peptides ranged from 0.78 (P9) to 0.94 (P7). The AUC for P1, P6, P7 and P8 were>0.90, indicating their high discriminatory ability for TB patients and PPD-/PPD+/PPD unknown subjects from endemic settings. The P values for difference between the NTBLD and TB patients ranged from 0.0073-<0.0001 for P1–P8; for P9 the P value was 0.7221.

The selection criteria for peptides to be evaluated with sera from larger cohorts of subjects included a) statistically significant difference between reactivity with the sera from TB and NTBLD patients; b) broad range of reactivity (reflectance values) with the sera from TB patients, and c). at least 60% sensitivity in TB patients. Based on these criteria, P1, P6 and P7 were selected for further studies (Fig 2A).

**Figure 3:**
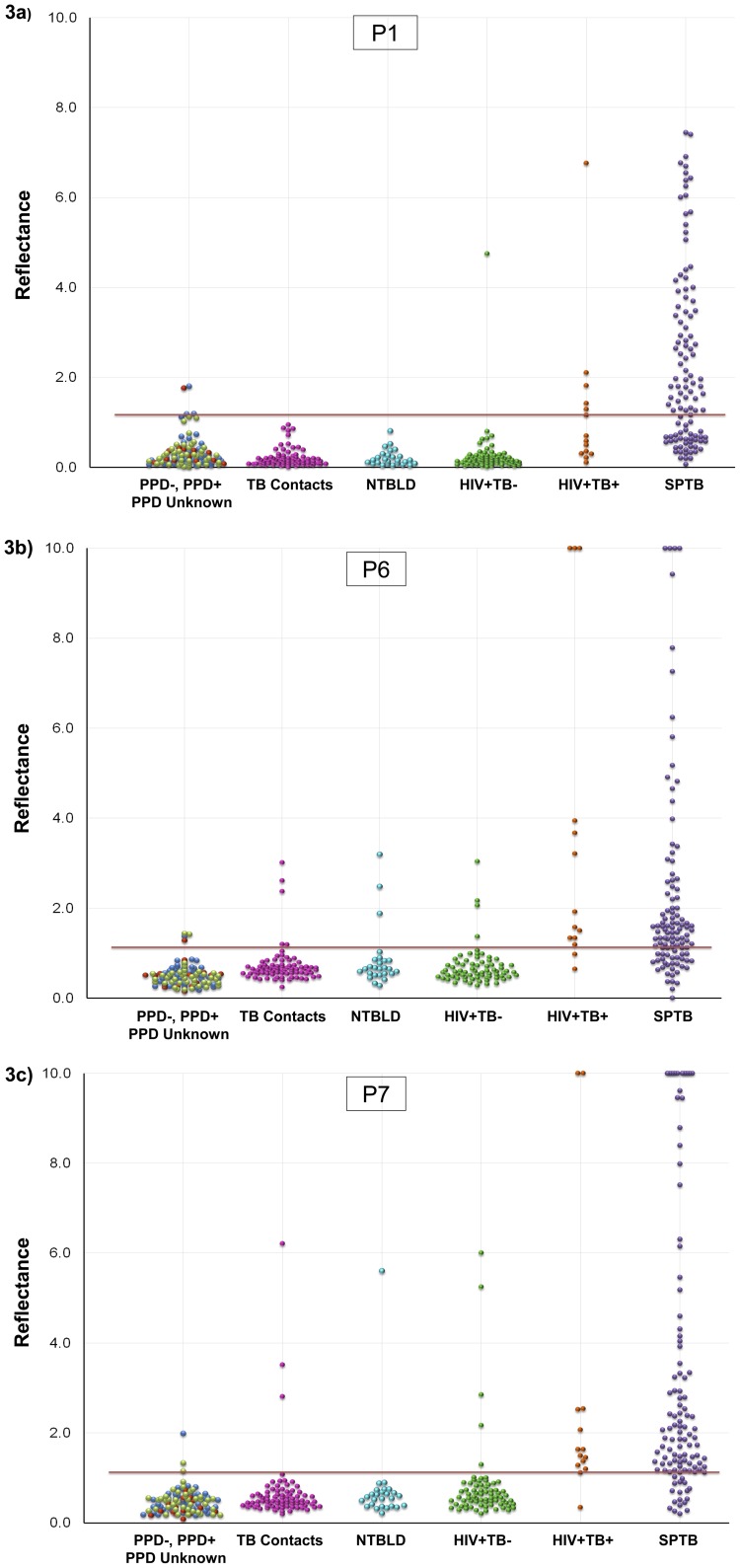
Reactivity of sera from an expanded cohort of subjects (n = 400) with selected BSA-peptide conjugates. Reflectance values obtained with sera from healthy subjects [PPD- (blue), PPD+ (light green), PPD unknown (red); n = 98], close and household contacts of infectious TB patients (pink; n = 80); non-TB lung disease patients [NTBLD (light blue); n = 26], HIV-positive, TB negative patients (green; n =  72); HIV-positive, smear positive TB patients (orange; n = 14) and non-HIV sputum smear-positive TB patients [SPTB (purple); n = 110] tested for reactivity to BSA-peptide conjugates (A) P1, (B) P6 and (C) P7 on lateral flow are plotted. Dashed lines indicate mean reflectance of the 98 healthy subjects plus 2.5 times the standard deviation which was used as cut-off to determine specificity in non-TB subjects and sensitivity in TB patients. Reflectance values exceeding 10.0 were plotted at 10.0.

**Figure 4:**
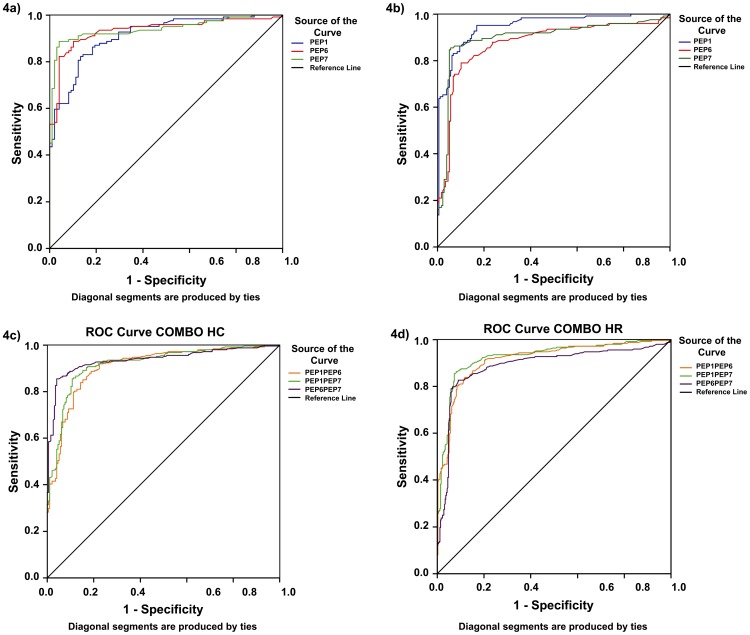
ROC curves representing the performance of P1, P6 and P7 BSA-peptide conjugates on lateral flow format. (A) ROC curves for reactivity of individual BSA-peptide conjugates with sera from healthy subjects and all smear-positive TB patients. (B) ROC curves for reactivity of individual BSA-peptide conjugates with sera from close/household contacts/NTBLD patients/HIV+TB- patients as reference standard, and all smear-positive TB patients. (C) ROC curves representing the additive performance of P1+P6, P1+P7 and P6+P7 conjugates for reactivity with sera from all smear-positive TB patients and healthy PPD-/PPD+/PPD-unknown subjects as reference standard. (D) ROC curves representing the additive performance of P1+P6, P1+P7 and P6+P7 conjugates for reactivity with sera from all smear-positive TB patients and from close/household contacts/NTBLD patients/HIV+TB- patients as reference standard.

Reactivity of BSA-peptide conjugates P1, P6 and P7 was tested with an expanded cohort comprising of sera from 98 healthy subjects (41 PPD-, 42 PPD+ and 15 PPD-unknown subjects). The mean reflectance obtained with these 98 sera plus 2.5 standard deviations was used as cut-off for evaluating reactivity of each BSA-peptide-conjugate with sera from 80 close and house-hold contacts of untreated TB patients, 26 NTBLD patients and 72 HIV+TB- patients (Fig 3). The reactivity of individual serum specimens with the BSA-peptide conjugates (P1, P6 and P7) on lateral flow is shown in Fig 3A–C. The sensitivity and specificity of antibody detection with each of the 3 peptides obtained individually and in combinations (additive) is shown in Table 2. Using the lateral flow format, the sensitivity of detection of antibodies for the 3 individual peptide-BSA conjugates in sera from 124 TB patients ranged from 58–82%. The specificity values with sera from 3 types of subjects were high. The overall specificity with the 178 TB-negative subjects at a higher risk of TB compared to the normal healthy population in TB endemic settings, ranged from 92–99%. Thus, sera from a large proportion of TB patients contained detectable antibodies to multiple peptides while sera from non-TB patients did not. ROC curves for the 3 peptides for the 124 TB patients with the 98 control group as reference (Fig 4A) or the 178 subjects at increased risk of TB (Fig 4B) were plotted. The AUCs of P1, P6 and P7 for all TB patients with the healthy PPD-/PPD+/PPD-unknown from TB-endemic countries were 0.91, 0.93 and 0.94 respectively (Fig 4A). Taking the close contacts and household contacts of TB patients, patients with NTBLD and the HIV+TB- patients as reference standard, the AUC values were 0.95, 0.875 and 0.898 for P1, P6 and P7 respectively (Fig 4B).

**Table 2:** Performance characteristics of peptides and peptide combinations for TB diagnosis.

Peptide	Sensitivity (95% CI)	Specificity (95% CI)
P1	58.1% (48.9, 66.9)	99.4% (96.9, 100.0)
P6	71.0% (62.1, 78.8)	92.1% (87.2, 95.6)
P7	82.3% (74.4, 88.5)	93.8% (89.2, 96.9)
P1+P6	86.3% (79.0, 91.8)	92.1% (87.2, 95.6)
P1+P7	91.9% (85.7, 96.1)	93.8% (89.2, 96.9)
P6+P7	87.1% (79.9, 92.4)	90.5% (85.2, 94.3)
P1+P6+P7	94.4% (88.7, 97.7)	90.5% (85.2, 94.3)

To determine if the additive reactivity of individual peptide-conjugates could enhance the detection of the anti-peptide antibodies, additive sensitivity with different peptide-BSA conjugates (tested individually) was calculated (Table 2). Additive reactivity with different combinations of two peptides provided sensitivities ranging from 86–91% and specificities ranging from 90–93.8%. The additive sensitivity with the 3 peptides (P1+P6+P7) provided 94% sensitivity in TB patients and specificity was maintained at 90.5% (Table 2). Taking the PPD-/PPD+/PPD-unknown subjects as the reference standard, the AUCs of P1+P6, P1+P7, and P6+P7 were 0.90, 0.919 and 0.937 respectively (Fig 4C). Taking the 178 and household contacts of TB patients, patients with NTBLD and the HIV+TB- patients as reference standard the AUC values were for the three peptide combinations were 0.915, 0.926 and 0.888 respectively (Fig 4D).

## Discussion

Genes for the three highly immunogenic proteins whose immunodominant epitopes have been used as peptides in these studies are present in the *M. tb* complex and all clinical isolates of *M. tb* sequenced so far but either absent in the non-tuberculous mycobacteria (*M. leprae, M. avium, etc*) that cause disease in humans, or the homologous proteins show limited identity. Our earlier studies in the ELISA format had demonstrated that antibodies to these individual immunodominant epitopes are detected in sera from at least 40% of the TB patients tested, but not in subjects with latent *M. tb* infection [7], [8]. Moreover, since a vast majority of the specimens included were from India, where BCG vaccination is given at birth, antibodies to these peptides are also absent in the vaccinated individuals. The current studies extend these findings and demonstrate that; a) anti-peptide antibodies can also be detected in the lateral flow format; b) these antibodies are absent not only in sera from subjects with latent *M. tb* infection and/or BCG vaccination but also in subjects who are at a high risk of current *M. tb* exposure and infection due to intimate contact with highly infectious, untreated TB patients; c) in sera from patients with NTBLD who clinically would be TB suspects, and d). in sera from HIV+ patients who do not have TB. Importantly, even at this early stage of development and optimization, combinations of just 2 or 3 of these immunodominant -BSA-peptide conjugates achieve high sensitivity (>90%) as well as specificity (>90%). The discriminatory ability of the peptides for TB patients and the populations from TB-endemic setting and at high-risk for TB, on a POC format is highly encouraging that development of a simple rapid POC test that can replace microscopy for use by TB control programs in the endemic settings and in peripheral settings will be possible.

Our earlier results with the recombinant proteins demonstrated that antibodies to PTRP and LipC were present in sera from 80–90% of the ∼50 of HIV+TB+ patients tested [7], [8]. While the number of HIV+TB+ patients whose sera have been included in the evaluation of reactivity with the BSA-peptide conjugates on the lateral flow format is small, and sera from larger cohorts will need to be examined, it is reassuring that the high sensitivity of anti-peptide antibody detection was obtained on the rapid format also. We (and others) have earlier provided evidence that the presence of antibodies to other *M. tb* antigens (Malate Synthase, MPT51) in sera from HIV+TB+ patients is independent of the CD4+ T-cell status of the patient [13], [14]. The HIV+TB- patients included in the current studies were all from the US, and on ART, and therefore at negligible risk of TB. The HIV+TB- cohort was deliberately not obtained from India, because studies from multiple TB-endemic countries have shown that ∼10–20% of the asymptomatic, ambulatory HIV+ patients in these settings have unrecognized TB, that is identified only by Intensive case finding [15]–[17]. Also antibodies to selected *M. tb* proteins have been reported in sera of asymptomatic HIV+ patients who progressed subsequently to symptomatic and bacteriologically proven TB within 2–6 months [13]. Because there are no diagnostic tests that can conclusively rule out asymptomatic, bacteriologically negative TB, inclusion of HIV+TB- patients from a TB-endemic setting would give false low specificity results.

The specificity in the subjects who are at an elevated risk for TB was only marginally lower compared to the healthy PPD-/PPD+ subjects. This is important because close, especially house-hold contacts of infectious TB patients are at a higher risk of TB, and although chest Xrays were performed, extensive investigations like bacterial cultures or NAATs (eg geneXpert), which would enable more accurate ruling-out of TB could not be used. Similarly, the NTBLD patients were diagnosed to have other lung diseases, but TB was not specifically ruled out in these patients [18]–[20]. Co-existence of TB and other lung diseases has often been reported, and the possibility of some of the sera that showed reactivity with the peptides were from patients with dual pulmonary disease cannot be ruled out. Moreover, the optimization of the POC test remains to be finalized, and it is also possible that modifications in the assay components will help to reduce any non-specific interactions that were observed.

Efforts to devise simple rapid POC tests for TB have been made for decades without success, and while>20 serological test were sold in TB-endemic countries for several years, recent WHO-sponsored meta-analysis of performance of these tests as well as the laboratory-based evaluation of 19 different tests by the WHO has clearly demonstrated their failure to accurately diagnose TB [21]–[24]. These results led to the WHO issuing a negative policy statement in 2011, recommending that the current serological tests not be used for diagnosis of TB [25]. The WHO policy also makes a strong suggestion that research on identification of new markers for devising a rapid POC test for TB be intensified. Some of the commercial rapid tests that failed to provide accurate results were based on use of total lysates or culture filtrates of *M. tb* which contain conserved bacterial proteins (heat shock proteins of *M. tb*, housekeeping proteins like the metabolic enzymes etc); others were based on recombinant proteins that were selected on the basis of their quantitative dominance in culture filtrates (Ag 85 complex, 38 kDa PhoS proteins), and yet others were proteins that were highly immunogenic in immunized animals or were selected simply because they were easily available [21]–[23]. In contrast, our approach is to first confirm that a). the proteins are highly immunogenic in TB patients but not in subjects with latent infection and/or BCG vaccination; b) the proteins are equally well-recognized by antibodies in HIV-TB+ and HIV+TB+ patients; c) the genes/proteins are *M. tb* complex specific and are present in all clinical isolates sequenced so far and d). either the genes/proteins are absent in other mycobacteria that cause disease in humans or the homologs have limited identity. Moreover, rather than include the full-length protein, only the highly immunodominant regions of these proteins are included as peptides to capture the antibodies.

The current results are based on studies conducted with only 9 of the 33 peptides, and inclusion of additional/alternate peptides to further enhance both the sensitivity and specificity of antibody detection is ongoing. While the final test will be evaluated for its performance in TB suspects, the strategy of including only those peptides that provide high sensitivity of antibody detection in TB patients as well as high specificity in individuals who are at a higher risk for TB or would clinically mimic TB enhances the ability to devise an accurate test. It is encouraging that even at this early stage of test development, both sensitivity and specificity>90% has been achieved. The WHO has defined the specifications that a rapid POC test must meet to be able to replace microscopy [1], [26]. The desired specifications are a sensitivity of>95% in sputum smear-positive TB patients,>60% in sputum smear-negative but culture positive TB patients, and a specificity of>98% [1], [26]. However, because of the high rates of patients who fail to return for the second (or third) sputum examination and/or for results of the sputum smears, which are required to initiate treatment, the WHO has defined the minimum specifications to be a sensitivity of>90% in sputum smear-positive patients, and a specificity of>95% [26]. Whether these specifications refer to microscopy as performed in the TB-endemic settings (smears made directly from the sputum and stained with ZN) or in low-burden settings (smears made with decontaminated and concentrated specimens) is not clearly stated. [5], [27], [28]. It is also not clear if the population of patients where the specificity of>95% [26] is to be achieved refers to TB suspects, or smear-negative TB suspects who fail to respond to the broad spectrum antibiotic treatment, or patients with presumptive TB [29], [30] These issues are difficult to address in TB where even with the liquid cultures, 10–20% of the patients have presumptive TB which cannot be confirmed bacteriologically and is diagnosed on the basis of response to TB treatment [29], [30]. Modeling suggests that a highly sensitive but not-highly specific POC could be useful as an effective screening test to identify suspects who need to be tested with the expensive diagnostic tests [31].

A recent survey of microscopy centers where the vast majority of the TB cases are diagnosed in the 22 high-burden TB-endemic countries has highlighted the lack of infrastructure (electricity, humidity, temperature control), basic equipment (biosafety hoods, water baths, incubators, centrifuges, gloves, masks), and personnel skills (pipetting, computer skills, etc) [27], [28]. Several new molecular tests that may replace the GXP are emerging [5], [32], but whether these can be implemented at the POC remains to be determined. In contrast, the simple POC test that is currently being developed by us, once fully optimized and validated, has the potential to be easily implemented in peripheral and remote settings like the microscopy centers.
